# Real-World Assessment of Pharmacokinetics, Clinical Outcomes, and Costs After Switching from Standard Half-Life to Extended Half-Life FVIII in Well-Controlled Hemophilia A Patients

**DOI:** 10.3390/hematolrep17050053

**Published:** 2025-10-17

**Authors:** Maria Choví-Trull, Juan Eduardo Megías-Vericat, Santiago Bonanad-Boix, Saturnino Haya-Guaita, Ana Rosa Cid-Haro, Marta Aguilar-Rodriguez, Tomás Palanques-Pastor, Javier Garcia-Pellicer, Jose Luis Poveda-Andrés

**Affiliations:** 1 Pharmacy Department, Hospital Universitari i Politècnic La Fe, 46026 Valencia, Spain; megias_jua@gva.es (J.E.M.-V.); tomas_palanques@iislafe.es (T.P.-P.); garcia_javpel@gva.es (J.G.-P.); 2 Hemostasis and Thrombosis Unit, Hospital Universitari i Politècnic La Fe, 46026 Valencia, Spain; bonanad_san@gva.es (S.B.-B.); haya_sat@gva.es (S.H.-G.); cid_ana@gva.es (A.R.C.-H.); 3 Fisioterapia en Movimiento, Grupo de Investigación Multiespecialidad (PTinMOTION), Universitat de Fisioterapia de València, 46010 Valencia, Spain; marta.aguilar@uv.es; 4 Management Department, Hospital Universitari i Politècnic La Fe, 46026 Valencia, Spain; poveda_josand@gva.es

**Keywords:** hemophilia A, pharmacokinetics, factor VIII, plasma half-life, polyethylene glycols, turoctocog alfa pegol

## Abstract

**Objective:** This study aimed to analyze pharmacokinetic and clinical parameters (bleeding rates and joint health) before and after switching from standard half-life (SHL) factor VIII (FVIII) to extended half-life (EHL) PEGylated turoctocog alfa pegol in patients with severe/moderate hemophilia A (HA) on prophylaxis, one year prior to and following the switch in a real-world setting. **Methods:** A single-center, comparative, observational, sequential, retrospective, multidisciplinary study was designed. The population pharmacokinetic parameters were estimated using the WAPPS-Hemo^®^ platform. The annualized bleeding rate (including total and joint bleeds), joint health (Hemophilia Joint Health Score), FVIII consumption, administration frequency, and treatment costs were analyzed. **Results:** Eight patients with severe (n = 7) or moderate (n = 1) HA on prophylaxis were included after switching to turoctocog alfa pegol. With this regimen, the median FVIII half-life was 16.8 (15.2–19.1) hours, the area under the curve (AUC) was 18,182 (12,879–21,214) IU·h/dL, and the incremental recovery was 2.2 IU/dL per (1.6–2.4) IU/kg. The patients required a median of 2.0 infusions per week (2.0–2.0), corresponding to a weekly consumption of 57.8 (54.2–61.1) IU/kg. Clinically, the prophylactic regimen was associated with fewer infusions per week, stable joint health, and a reduction in overall treatment costs. **Conclusions:** Prophylaxis with turoctocog alfa pegol provided the expected pharmacokinetic profile of an EHL-FVIII concentrate, enabled a lower infusion frequency, and was linked to a decreased treatment burden and cost while maintaining joint health.

## 1. Introduction

Hemophilia A (HA) is a congenital bleeding disorder characterized by a deficiency of factor VIII (FVIII), leading to impaired hemostasis and a high risk of bleeding—particularly joint bleeds—resulting in a functional deterioration and a reduced quality of life. Prophylaxis with FVIII concentrates has been the standard therapeutic strategy for preventing bleeding and preserving joint health [1]. However, standard half-life (SHL) products require frequent infusions, which may affect adherence and compromise their long-term effectiveness [2].

In recent years, the development of extended half-life (EHL)–FVIII products has represented a significant advancement, allowing for the maintenance of protective plasma levels with less frequent dosing [3]. Turoctocog alfa pegol (Esperoct^®^) was approved in 2019 and is administered at a recommended dose of 50 IU/kg every 4 days, with subsequent individualization based on bleeding episodes. Its pharmacokinetic properties include a mean half-life of approximately 19–24 h and predictable incremental recovery [4,5].

However, the data on its performance in routine clinical practice remain limited, and there is a growing need for real-world studies to validate these findings in more heterogeneous populations and diverse healthcare settings. Specifically, it is relevant to assess whether switching from an SHL product to turoctocog alfa pegol translates into tangible clinical and pharmacokinetic benefits, such as increased drug exposure, reduced treatment burden, or improved bleeding control.

This study aims to analyze, in a real-world setting, the differences in pharmacokinetic and clinical parameters after switching from an SHL-FVIII to turoctocog alfa pegol in patients with moderate or severe HA on prophylaxis. The findings will provide evidence on its utility in treatment individualization and its potential impact on therapeutic efficiency.

## 2. Materials and Methods

We conducted a single-center, retrospective, sequential, observational, and multidisciplinary study approved by the local Clinical Research Ethics Committee on 26 January 2022 (HUF-PKFVIII-2022-01), in accordance with the principles of the Declaration of Helsinki. This study was performed at Hospital Universitario y Politécnico La Fe (Valencia, Spain), following routine clinical practice standards, between January 2022 and December 2023, and no special treatment interventions were required.

### 2.1. Patient Population

Inclusion criteria were:

(1) Patients with severe (<1 international units (IU)/deciliter (dL)) or moderate (1–5 IU/dL) HA on prophylactic treatment with SHL-FVIII who switched to EHL-FVIII turoctocog alfa pegol;
(2) Patients registered in the WAPPS-Hemo^®^ database [6] with records of at least 3–4 FVIII level measurements and individualized pharmacokinetic (PK) profiles. Patients with <1 year of follow-up data before and after switching, or with missing records, were excluded.

### 2.2. Study Periods

Two one-year periods were analyzed: 1 year of SHL-FVIII treatment before the switch and 1 year after the switch to turoctocog alfa pegol. The initial dosing regimen was determined according to the prescribing information (50 IU/kg every 4 days) [7] and subsequently individualized using WAPPS-Hemo^®^ PK profiles and clinical criteria. Breakthrough bleeds were managed on demand according to WFH guidelines [1]. The aim of switching from SHL-FVIII to EHL-FVIII was to improve bleeding control and patient quality of life.

### 2.3. Pharmacokinetic Parameters

Data were collected from electronic medical records and the WAPPS-Hemo^®^ database, both of which are prospectively updated, ensuring data reliability despite the retrospective design. FVIII levels were measured using a one-stage clotting assay (OSA) with Actin FS activator and FVIII-deficient plasma (Siemens^®^). WAPPS-Hemo^®^ was used with 3–4 blood samples collected according to the International Society on Thrombosis and Hemostasis recommendations for Bayesian estimation of individualized PK profiles: pre-dose, 4–8 h, 16–28 h, and 40–60 h post-infusion for SHL-FVIII, with an additional sample at 60–84 h for PEG-FVIII [8].

The PK and clinical variables analyzed were: t_1/2_; AUC; peak level (PL); FVIII concentration at 24, 48, and 72 h (C24/C48/C72); time to reach FVIII levels of 5%, 2%, and 1% (T5%/T2%/T1%, representing the time elapsed after infusion until plasma FVIII activity falls below a specific threshold); and target trough level (TL, the minimum plasma FVIII concentration aimed to be maintained immediately before the next infusion, as the therapeutic goal was to ensure continuous hemostatic protection). The AUC was obtained from data provided by McMaster University. All PK parameters were calculated using the WAPPS-Hemo^®^ application, which employed the application’s clinical calculator to obtain PL, C24/C48/C72, and TL from the individualized PK profiles. The t_1/2_ and AUC ratios were calculated, and incremental recovery (IR) was determined for both SHL and EHL concentrates in each patient as the ratio of the difference between FVIII plasma concentration (peak level–trough level), divided by the infused dose (IU/kg).

### 2.4. Clinical Outcomes and Resource Use

Effectiveness was assessed using the annualized bleeding rate (ABR) and annualized joint bleeding rate (AJBR). The percentage of patients with zero bleeds during the study period, the target joints, and joint health score (using the Hemophilia Joint Health Score (HJHS)) were also evaluated. To estimate FVIII consumption, the number of weekly doses and the dose/kilogram (kg)/week were calculated, enabling the estimation of the cost per year per patient to provide economic data and assess resource utilization with the use of PEGylated factors. Annual cost was calculated as (IU/kg per week × body weight × price per IU).

### 2.5. Statistical Analysis

Statistical analysis was performed using R Statistical Software (version 4.3.3; 29 February 2024). The Wilcoxon signed-rank test was used to compare variables between the two study periods. A *p*-value < 0.05 was considered statistically significant. Results of quantitative variables were expressed as median and interquartile range (IQR) and qualitative variables as absolute number and percentage.

## 3. Results

The study included eight patients diagnosed with severe (n = 7)/moderate (n = 1) HA on prophylaxis who switched from SHL-FVIII (turoctocog alfa (Novoeight^®^) (n = 5), lonoctocog alfa (Afstyla^®^) (n = 1), or human coagulation FVIII/von Willebrand factor (Fandhi^®^) (n = 2)) to PEGylated EHL-FVIII (turoctocog alfa pegol (Esperoct^®^)). Two one-year periods (before and after the switch) were analyzed for each patient.

All the patients were adult males with a median age of 47.5 (42.8–54.5) years, a body weight of 67.5 (59.8–68.9) kg, and a body mass index (BMI) of 22.6 (22.5–23.3) kg/m^2^. Regarding the demographic characteristics, most of the patients were white (n = 6, 75.0%), with one Hispanic patient and one Black patient. As for the severity of HA, the majority had severe HA (n = 7, 87.5%), and one patient had moderate HA (12.5%). Table 1 summarizes the patients’ baseline characteristics.

The frequency of SHL infusions was three (2.8–3) times per week, while with the EHL factor, it was adjusted to two times per week in all patients. The median dose per administration was 30.1 (27.1–33.5) IU/kg for SHL and 28.9 (27.1–30.5) IU/kg for turoctocog alfa pegol, corresponding to weekly doses of 86.4 (64.3–93.2) and 57.8 (54.2–61.1) IU/kg/week, respectively. This translated into a 33.1% reduction in the weekly administered dose, avoiding a median of 52 injections per patient per year (33.3%). The median IR for SHL was 2.9 (2.2–3.2), while for EHL it was 2.2 (1.6–2.4).

**Figure 1:**
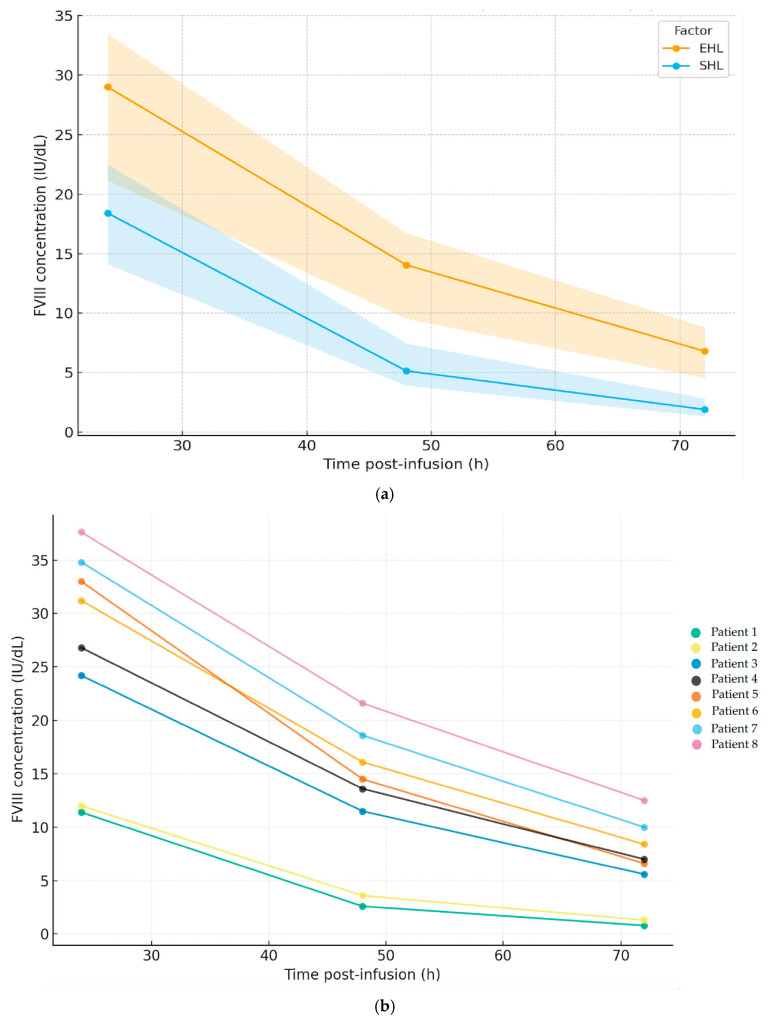
FVIII concentration at 24, 48, and 72 h with SHL and EHL (turoctocog alfa pegol) versus time post-infusion; (**a**) median and IQR; (**b**) individual EHL-FVIII profile per patient (n = 8). Abbreviations: SHL: standard half-life product; EHL: extended half-life product. Patients 1 and 2 showed lower levels at all time points, consistent with their shorter half-lives on both SHL and EHL; however, these reduced FVIII profiles did not translate into increased bleeding.

The target TL with turoctocog alfa pegol was 1–3% in two (25.0%) patients, 3–5% in one (12.5%) patient, and 5–15% in five (62.5%) patients, in contrast to the TLs achieved with the SHL factors, which showed an inverse distribution: 1–3% in five patients (62.5%), 3–5% in one (12.5%) patient, and 5–15% in two (25.0%) patients. However, the difference between the median TLs for SHL versus EHL did not reach statistical significance. Regarding the remaining PK variables, the use of PEG-FVIII was associated with statistically significant differences in most parameters (*p* < 0.05) compared to SHL, except for the plasma PL, which was lower post-switch (66.0 (53.5–71.5) IU/dL). The AUC reached was 18,182 (12,879–21,214) (IU·h)/liter (L). The time to reach factor levels of 1%, 2%, and 5% were 150.1 (126.1–170.1), 116.6 (97.6–132.6), and 82.6 (67.3–93.8) h, respectively, and for turoctocog alfa pegol, the elimination t1/2 was superior to that observed with the SHL factors (22.9 h versus 13.8 h). The FVIII concentrations were 29.0 (21.2–33.5), 14.1 (10.5–16.7), and 6.8 (4.5–16.7) IU/dL at 24, 48, and 72 h, respectively. Figure 1 shows the time-course of FVIII plasma concentrations (median, IQR) comparing the SHL concentrates with turoctocog alfa pegol (a) and the individual EHL-FVIII profile per patient (b).

As for changes in the clinical outcomes, the bleeding episodes were well controlled, maintaining a post-switch median ABR and AJBR of 0.0 (0.0–0.0), although without statistical significance. The number of patients with zero total and joint bleeds was seven in both cases (87.5%), with four bleeds avoided in three (37.5%) patients. The HJHS score obtained after the switch was 32.0 (19.0–39.0), comparable to a baseline HJHS of 32.5 (19.0–39.0). Table 2 summarizes the PK parameters and the analyzed variables.

The median annual cost per patient decreased from EUR 99,694 (98,544–201,052) with SHL to EUR 83,381 (62,597–83,442) with turoctocog alfa pegol, yielding an estimated savings of EUR 23,408 (15,096–168,544) per patient per year and EUR 994,100 for the cohort.

## 4. Discussion

This real-world case series analyzed the impact of switching from SHL factors to the PEGylated EHL factor turoctocog alfa pegol in patients with moderate/severe HA, analyzing PK parameters, clinical outcomes, and associated costs. As expected with EHL products, the half-life of FVIII activity was prolonged compared to the SHL concentrates, allowing a less frequent administration. This led to a reduced treatment burden and substantial cost savings. Few real-world studies have assessed these differences, but our findings are consistent with those reported in pivotal trials [4,5], with some particularities.

Switching to turoctocog alfa pegol was associated with a significant increase in t_½_ (22.9 h versus 13.8 h), consistent with the pharmacokinetic profile of EHL products, which allowed a reduction in the infusion frequency from three to two times per week. This increase in t_½_ is consistent with previous studies reporting a half-life of approximately 19–24 h for this product [4,5]. The first-in-human trial of turoctocog alfa pegol in 26 previously treated patients with severe HA (84.6% Caucasian) reported a T1% of 6.5 (3.6–7.9) days and a t_½_ of 19.0 (11.6–27.3) h after a 50 IU/kg dose, representing a 1.6-fold increase over the patients’ previous factor treatments [4]. Similar results were observed in our cohort, who received a median weekly dose of 57.8 (54.2–61.1) IU/kg, achieving a median T1% of 6.3 (5.3–7.1) days and a t_½_ of 22.9 (18.2–24.9) h. However, it should be noted that the previous trial used a chromogenic assay for analysis, whereas our study employed an OSA, which could partly explain the observed differences.

Regarding clinical efficacy, in the pivotal PATHFINDER2 trial (NCT01480180) [5], a median ABR of 1.3 (0.0–4.6) was reported, representing the lowest rate among PEGylated EHLs included in the systematic review by Graf et al. (2020) [9]. In our cohort, the ABR was 0.0 (0.0–0.0). This well-controlled status of the cohort (no inhibitors, few bleeding episodes, and only one target joint) limited the possibility of demonstrating a clinical benefit beyond an infusion frequency reduction, as a high proportion of the patients in the pivotal trial had target joints.

The dosing regimen for turoctocog alfa pegol in our cohort was 57.8 (54.2–61.1) IU/kg/week, administered twice weekly, which is in line with the pivotal trial regimen (50 IU/kg every four days). However, the extension study of the PATHFINDER2 trial demonstrated that a once-weekly regimen of 75 IU/kg provided a comparable clinical efficacy while maintaining similar bleeding rates. This reduced dosing frequency is a potential advantage for patients and should be considered in future dose adjustments [5], as this is one of the most valued aspects by patients [10]

In our study, a higher number of patients achieved a higher target TL, with 62.5% of the patients falling within a target trough level of 5–15%, compared to only 25% before the switch, indicating increased protection against bleeding with the PEGylated EHL factor. The baseline HJHS score of 32.5 (19.0–39.0) reflected pre-existing joint damage in most of the patients, likely accumulated before the initiation of effective prophylaxis. After the switch, the HJHS remained stable at 32.0 (19.0–39.0), indicating that the new treatment did not alter the joint status but may have contributed to the prevention of further bleeding-related complications. Nonetheless, given the restricted number of events, any conclusions on the clinical efficacy should be interpreted with caution, so this study mainly provides supportive PK data and information on dosing frequency and cost.

These results should be interpreted in light of the study population, which consisted mainly of patients with severe hemophilia A and no target joints who may achieve adequate protection with less frequent dosing. A further evaluation of extended dosing intervals, including once-weekly prophylaxis, is warranted in this subgroup.

Regarding the limitations of this study, first, the sample size in this case series was relatively small, so the results should be interpreted with caution and therefore cannot be generalized to the broader hemophilia population; however, this size may still be relevant, considering previously published studies and the fact that HA is a rare disease. Second, the external validity of this single-center study is limited, and its applicability to other HA cohorts may not be appropriate. Third, despite the use of a sequential design in which each patient served as their own control, the heterogeneity among the HA patients (e.g., age, weight, joint status, von Willebrand factor levels) could influence their bleeding scores and the PK estimates. Finally, patient-reported outcomes (adherence, satisfaction, quality of life, etc.) were not planned to be collected at the time of the PEG-FVIII switch at our center. The adherence to prophylaxis was also not formally measured. Nevertheless, according to the pharmacy dispensing records and the physician notes, no issues related to treatment compliance were identified during the follow-up. However, we acknowledge that the absence of patient-reported adherence measures is an additional limitation.

## 5. Conclusions

This study provides novel real-world pharmacokinetic data for turoctocog alfa pegol. Significant improvements were observed in both the t_½_ (>1.3) and the AUC (>1.25), fulfilling the EHL-FVIII thresholds and enabling fewer weekly infusions. These PK findings add valuable evidence for clinical practice, supporting a reduced treatment burden in hemophilia A. Although clinical benefits and cost savings were noted, the interpretation is limited by the small sample size.

## Figures and Tables

**Table 1:** Baseline characteristics of the patients included in this study.

Parameters (Median, IQR)	Patients (n = 8)
Age (years), median (IQR)	47.5 (42.8–54.5)
Weight (kg), median (IQR)	67.5 (59.8–68.9)
BMI (kg/m^2^), median (IQR)	22.6 (22.5–23.3)
Height (cm), median (IQR)	172.5 (167.0–174.2)
Pre-switch HJHS, median (IQR)	32.5 (19.0–39.0)
Target joint, n (%)	0.0 (0.0)
Pre-switch ABR, median (IQR)	0.0 (0.0–0.0)
Pre-switch AJBR, median (IQR)	0.0 (0.0–0.0)
HA severity, n (%) Moderate	1 (12.5)
Severe	7 (87.5)
Race, n (%) White	6 (75.5%)
Hispanic	1 (12.5%)
Black	1 (12.5%)
Blood group, n (%) A	1 (12.5)
B	2 (25.0)
AB	3 (37.5)
0	1 (12.5)
Unknown	1 (12.5)
History of inhibitors, n (%)	0 (0.0)

ABR: annual bleeding rate; AJBR: annual joint bleeding rate; BMI: body mass index; cm: centimeter; HJHS: Hemophilia Joint Health Score; IQR: interquartile range; kg: kilogram; m: meter.

**Table 2:** PK parameters and analyzed variables.

PK Parameters and Analyzed Variables	SHL Factors (n = 8)	Turoctocog Alfa Pegol (n = 8)	*p*-Value
Treatment data			
Dose (IU/kg/week), median (IQR)	86.4 (64.3–93.2)	57.8 (54.2–61.1)	0.036
Weekly frequency, median (IQR)	3.0 (2.8–3.0)	2.0 (2.0–2.0)	0.020
PK parameters			
Peak level (IU/dL), median (IQR)	79.0 (72.0–90.3)	66.0 (53.5–71.5)	0.039
C24 (IU/dL), median (IQR)	18.4 (14.1–22.5)	29.0 (21.2–33.5)	0.008
C48 (IU/dL), median (IQR)	5.2 (3.9–7.5)	14.1 (9.5–16.7)	0.014
C72 (IU/dL), median (IQR)	1.9 (1.3–2.8)	6.8 (4.5–8.8)	0.014
TL (%), median (IQR)	2.8 (1.7–3.7)	5.2 (3.7–7.5)	0.078
T5% (h), median (IQR)	48.4 (43.1–57.5)	82.6 (67.3–93.8)	0.008
T2% (h), median (IQR)	70.2 (61.8–83.1)	116.6 (97.6–132.6)	0.008
T1% (h), median (IQR)	91.4 (79.9–114.0)	150.1 (126.1–170.1)	0.016
t_½_ (h), median (IQR)	13.8 (12.0–15.4)	22.9 (18.2–24.9)	0.008
AUC ((IU·h)/L), median (IQR)	10,879 (8816–13,069)	18,182 (12,879–21,214)	0.023
Clinical variables			
ABR, median (IQR)	0.0 (0.0–1.0) *	0.0 (0.0–0.0) *	0.174
AJBR, median (IQR)	0.0 (0.0–0.3)	0.0 (0.0–0.0)	0.3711
HJHS, median (IQR)	32.5 (17.5–41.3)	32.0 (19.0–39.0)	0.7835

ABR: annual bleeding rate; AJBR: annual joint bleeding rate; AUC: area under the curve; dL: deciliter; h: hours; HJHS: Hemophilia Joint Health Score; IQR: interquartile range; IU: international units; kg: kilogram; L: liter; PK: pharmacokinetics; t_½_: half-life time; T5%, T2%, T1%: time to reach FVIII levels of 5%, 2%, and 1%; TL: target trough level; C24, C48, C72: FVIII concentration at 24, 48, and 72 h. * Total number of bleedings with SHL (n = 6) vs. EHL (n = 2): 4 bleeding events [SHL (Patient 1: 1 bleeding, Patient 2: 1 bleeding, Patient 3: 4 bleedings), EHL (Patient 1: 0 bleedings, Patient 2: 0 bleedings, Patient 3: 2 bleedings)].

## Data Availability

The original contributions presented in this study are included in the article. Further inquiries can be directed to the corresponding author.

## Abbreviations

The following abbreviations are used in this manuscript:

ABR	annualized bleeding rate
AJBR	annualized joint bleeding rate
AUC	area under the curve
BMI	body mass index
EHL	extended half-life
FVIII	factor FVIII
H	hours
HA	hemophilia A
HJHS	Hemophilia Joint Health Score
IQR	interquartile range
IU	international units
OSA	one-stage clotting assay
PEG	polyethylene glycol
PK	pharmacokinetic
PL	peak level
SHL	standard half-life
t_½_	plasma half-life
T1%	time to reach 1% FVIII levels
T2%	time to reach 2% FVIII levels
T5%	time to reach 5% FVIII levels
TL	trough level
TL24	trough level at 24 h
TL48	trough level at 48 h
TL72	trough level at 72 h
